# Validation of the Cancer Health Literacy Test-30 for Populations Without Cancer

**DOI:** 10.3928/24748307-20180207-01

**Published:** 2018-04-12

**Authors:** Levent Dumenci, Robin K. Matsuyama, Daniel L. Riddle, Laura Cartwright, Laura A. Siminoff

## Abstract

**Background:**

Cancer incidence continues to be common and highly consequential for future cancer patients, family members, and other untrained caregivers. Because low health literacy increases the likelihood of poor health outcomes, those with low health literacy would benefit from interventions to improve cancer health literacy.

**Objective:**

Our study was designed to address the need for measuring cancer health literacy in populations without cancer.

**Methods:**

The Cancer Health Literacy Test-30 (CHLT-30) was psychometrically tested on 512 people without a cancer diagnosis.

**Key Results:**

In this population without cancer, the CHLT-30 had strong psychometric properties including unidimensional measurement structure, high reliability, and invariant measurement between gender, race/ethnicity, and educational-attainment groups.

**Conclusion:**

These results support the use of the CHLT-30 to measure cancer health literacy in research studies of family members of people with cancer or the general public. The potential for using the CHLT-30 in clinical practice to assess the need for education for general patient and cancer patient populations is a future direction. **[*HLRP: Health Literacy Research and Practice*. 2018;2(2):e58–e66.]**

**Plain Language Summary:**

Psychometric evidence suggests that the CHLT-30, originally developed for cancer patients, can be used to measure cancer health literacy of individuals with no history of cancer as well.

Health literacy is commonly conceptualized as the ability to seek, use, and perform knowledge-based tasks necessary to make health-related decisions in various settings such as homes, hospitals, and clinics (Kindig, Panzer, & Nielsen-Bohlman, 2004; Nutbeam, 1998; Sorensen et al., 2012). People with limited health literacy experience difficulties in making healthy life choices and effectively managing their own health, as well as having more frequent general hospital and emergency department visits, lengthier hospital stays, and higher rates of disease occurrence than do those with adequate health literacy (Baker, Parker, & Williams, 1998; DeWalt, Berkman, Sheridan, Lohr, & Baker, 2004; Paasche-Orlow & Wolf, 2007). The human and financial costs of limited health literacy are considerable in the United States (Howard, Gazmararian, & Parker, 2005) and abroad (Eichler, Wieser, Brugger, 2009). This is especially true for people with chronic and life-threatening diseases such as cancer (Baker et al., 2007; Cavanaugh et al., 2010; Omachi, Sarkar, Yelin, Blanc, & Katz, 2013; Song et al., 2012). These costs are not isolated to those suffering from chronic disease, but extend to family members and, especially, patient caregivers.

The American Cancer Society estimates that nearly 1.7 million people were diagnosed with a new cancer and 600,920 people died from cancer in 2017 (American Cancer Society, 2018). Moreover, the use of preventive health care services, including cancer screening, is essential for early detection and requires a health-literate population for decreasing cancer mortality (Davis et al., 2001; Lindau et al. 2002; Scott, Gazmararian, Williams & Baker, 2002). Therefore, cancer health literacy is a critical issue among the general population. Specific instances where cancer health literacy for people not diagnosed with cancer would be valuable include population surveys on cancer health literacy, research involving cancer health literacy of family and untrained caregivers, and cancer health literacy of people receiving cancer screening and diagnostic testing for cancer. We found no instrument to measure cancer health literacy in populations without cancer.

The Cancer Health Literacy Test-30 (CHLT-30; Dumenci et al., 2014) is a 30-item test developed to specifically measure cancer health literacy of people with cancer. The instrument has 30 multiple choice items that cover knowledge, skill, numeracy, navigation, and their synthesis by adopting the ability definition of the cancer health literacy construct. Disease-specific health literacy tests such as the CHLT-30 capitalize on more fine-grained content coverage than general health literacy tests and measure health literacy rather than the ability to read or write. The instrument has broad content coverage, high reliability (Cronbach alpha: 0.88–0.92), strong external validity (e.g., predicting self-confidence in engaging in health decisions), and invariant measurement properties between gender and race/ethnicity groups (Dumenci et al., 2014). The standardization sample of the CHLT-30 included only people with a history of cancer, limiting inferences to persons with cancer.

Given the utility of measuring cancer health literacy of people with no history of cancer and the psychometric strengths of the CHLT-30 in cancer populations, the primary objective of this study was to evaluate the psychometric properties of the CHLT-30 in a large sample of people without cancer. If the CHLT-30 can be applied with high validity to populations without cancer, the instrument could be used to measure and monitor cancer health literacy in general healthy and at-risk populations, help inform health policy challenges, and evaluate intervention effectiveness for a variety of stakeholders.

## Method

### Participants

Participants (*N* = 512) were people with no history of a cancer diagnosis. The sample consisted of 315 (61.5%) women and 197 (38.5%) men. The sample was 64.1% African American, 35.2% White, and 0.7% other (two Asian, one multiracial, and one unidentified race). Participant ages ranged from age 19 to 85 years (*M* = 49; standard deviation [*SD*] = 15). Educational attainment was as follows: less than high school diploma (15.2%), General Educational Development (GED) certificate (4.7%), high school diploma (20.1%), some college (22.7%), associate/technical degree (8.4%), bachelor's degree (17.%), and beyond a bachelor's degree (11.9%). Although no one had been diagnosed with cancer, one-third of participants (36.1%) reported they had taken care of someone with cancer.

### Procedures

The target sample was from the general population. Participants were recruited from primary care clinics, community centers, churches, and health fairs throughout the state of Virginia. Eligible participants reported no history of a cancer diagnosis, were English speaking, and age 18 years or older. To ensure comparability, the procedure employed in the previously reported study on persons with cancer (Dumenci et al., 2014) was replicated in the current study. The CHLT-30 was administered via a hand-held touch-screen device that also read the test items aloud. This administration procedure ensures that the CHLT-30 scores are not confounded with reading ability. The study protocol was approved by the Virginia Commonwealth University's Institutional Review Board.

### Measures

The CHLT-30 was administered along with the Rapid Estimate of Adult Literacy in Medicine (REALM; Davis at al., 1991), a word pronunciation test commonly used as a substitute to measure general health literacy (Dumenci, Matsuyama, Kuhn, Perera, & Siminoff, 2013). The REALM was administered to validate the CHLT-30 scores in predicting engagement in health decisions. Making appropriate health decision is explicitly stated as the outcome of health literacy in the Institute of Medicine report (Kindig, Panzer, & Nielsen-Bohlman, 2004). A four-item questionnaire involving two positively and two negatively worded items with a 1-to-5 Likert scale response format was used to measure self-confidence in engaging in health decisions (Dumenci et al., 2014).

### Analysis

The proposed measurement structure of a unidimensional latent variable model for the CHLT-30 was tested using the diagonally weighted least squares estimator, a distribution-free method, to account for binary item response distribution. The chi-square test of exact fit, root mean square error of approximations (RMSEA) and 90% confidence interval (CI) around the RMSEA, comparative fit index (CFI), and Tucker-Lewis index (TLI) were used to assess the data-model consistency using Hu-Bentler criteria (Hu & Bentler, 1999). In addition to the total sample, the model was tested separately for gender, race/ethnicity (Black and White), and educational attainment (≤ high school and > high school). The standardized factor loadings from the one-factor model were supplemented with the percent-correct item responses, item-corrected total correlations, as well as the item difficulty and discrimination parameters estimated from the two-parameter logistic (2-PL) Item Response Theory model (Embretson & Reise, 2000) to assess the performance of 30 items as indicators of the latent cancer health literacy construct. Results from the 2-PL were also used to estimate item and total test information curves to assess the measurement precision along the latent trait continuum. Reliability was estimated from Cronbach's alpha (Cronbach, 1951; *N* = 512), McDonald's omega (McDonald, 1999; *N* = 512), and a 2-week (*n* = 51) and 6-month (*n* = 25) test-retest correlation. Measurement invariance was tested between gender, race/ethnicity, and educational attainment groups using multigroup confirmatory factor analysis (Meredith, 1993). Once the measurement invariance was established, the equality of group latent means was tested using chi-square difference test and differences in RMSEA, CFI, and TLI (Millsap, 2011). External validity of the CHLT-30 was tested using structural equation modeling (SEM). The CHLT-30 scores were used to predict the self-confidence about engaging in health decisions as measured with a 4-item scale (Dumenci et al., 2014) after taking into account a general health literacy as measured by REALM (Davis et al., 1991), education, ethnicity, income, gender, and age. Mplus software (version 8; Muthén & Muthén, 2017) was used in the analyses.

## Results

### Measurement Model

**Figure 1.**
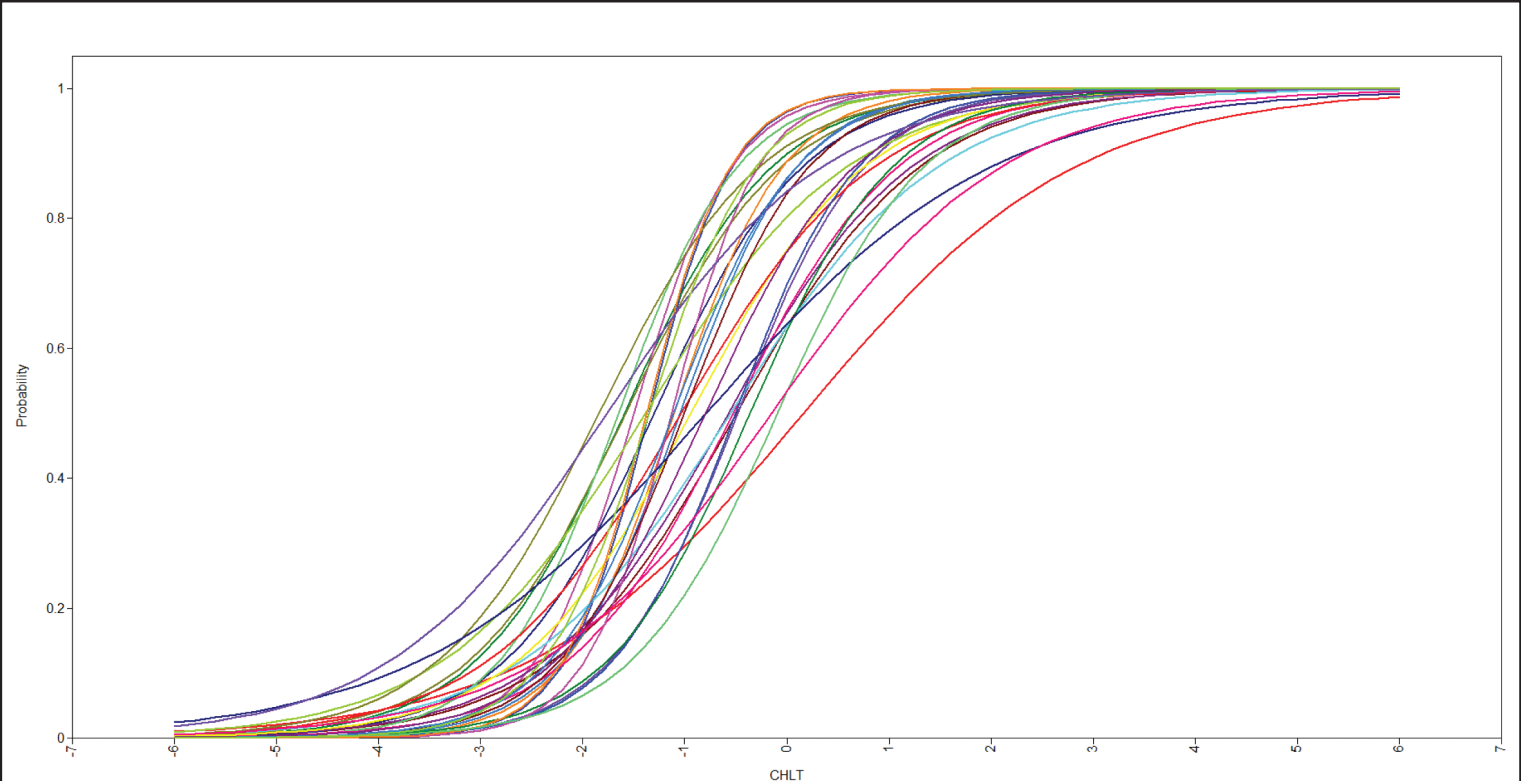
Item information curves from the two-parameter logistic model. CHLT = Cancer Health Literacy Test.

Model fit indices from a one-factor model specific to gender, race/ethnicity, and educational attainment groups and the combined sample are provided in **Table 1**. Fit indices strongly supported the unidimensionality of the cancer health literacy trait as measured by the CHLT-30. Item parameters are given in **Table 2**. All 30 items were relatively easy as indicated by high percent-correct responses (*M* = .72; median = .75; range: .47–.88) and all but one negative item difficulty parameter (*M* = −1.07; median = −1.12; range: −2.22–0.19). All standardized factor loadings were large (*M* = .58; median = .59; range: .36–.72) and significant (*p* < .001); item-corrected total correlations were significant (*p* < .001) and moderate in magnitude (*M* = .40; median = .41; range: .26–.52); item discrimination parameters were large (*M* = .73; median = .73; range: .38–1.04) and significant (*p* < .001). Consistent with this set of item parameters, all 30-item characteristic curves had sharp slopes (**Figure 1**). Taken together, all 30 items performed well as indicators of the continuous latent trait of cancer health literacy.

### Reliability

**Figure 2.**
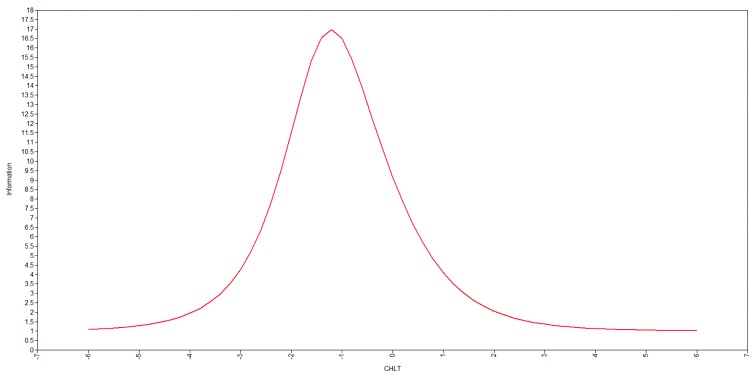
Test information curve from the two-parameter logistic model. CHLT = Cancer Health Literacy Test.

The CHLT-30 is a reliable measure of cancer health literacy as indicated by Cronbach's alpha internal consistency reliability of .87, McDonald's omega reliability of .94, 2-week test-retest reliability of .91, and 6-month test-retest stability of .93. The CHLT-30 test-retest mean scores (Mt1, Mt2) were not significantly different for the 2-week (Mt1 = 24.51; Mt2 = 24.51; *p* > .10) and 6-month (Mt1 = 19.16; Mt2 = 19.52; *p* > .10) intervals. From the test information curve estimated from the 2-PL (**Figure 2**), the CHLT-30 scores provide the most precise measurement at approximately 1 *SD* below the average score. These results indicate that the CHLT-30 is most sensitive to detecting small differences among people with low levels of cancer health literacy.

### Measurement Invariance

The measurement invariance test results appear in **Table 3**. Comparisons between gender, race/ethnicity, and educational attainment groups all supported the scalar invariance property of the CHLT-30, indicating that the cancer health literacy construct measured by the instrument refers to the same construct across observed groups by effectively ruling out the measurement bias interpretation of test scores. The difference test between the latent means indicated that men and women did not differ significantly (*p* > .10). However, Whites scored higher than Blacks (*p* < .001), and people with high levels of education (above high school) scored higher than those with low levels of education (high school or less; *p* < .001). The distributions of raw CHLT-30 scores were as follows: men: *M* = 20.77 (*SD* = 6.31); women: *M* = 22.05 (*SD* = 6.31); non-Hispanic Blacks: *M* = 19.20 (*SD* = 5.34); non-Hispanic Whites: *M* = 25.78 (*SD* = 4.62); ≤high school education: *M* = 18.02 (*SD* = 4.92); >high school education: *M* = 24.59 (*SD* = 4.54).

### External Validation

**Figure 3.**
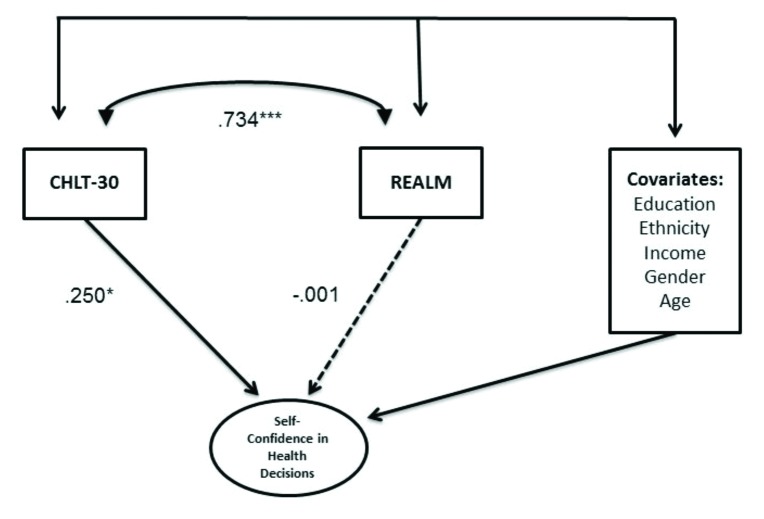
External validation of the CHLT-30 with self-confidence about engaging in health decisions. CHLT-30 = Cancer Health Literacy Test-30; REALM = Rapid Estimate of Adult Literacy in Medicine. * *p* < .05. *** *p* < .001.

The CHLT-30 scores were used to predict self-confidence in making health decisions, represented as a latent variable using two positively and two negatively worded items on a 4-point Likert-type response format, controlling for gender, race/ethnicity (White vs. Black), income, education, and general health literacy as measured by the REALM. The model fit well: *X*^2^ = 40.66; *df* = 23; *p* = .039; RMSEA = .039 (90% CI [.018, .058]); CFI = .894; and TLI = .844). The graphical representation of the SEM and standardized parameter estimates appear in **Figure 3**. The CHLT-30 was a significant predictor of self-confidence in engaging in health decisions (β = .25; *p* < .05). The REALM scores, however, were not significantly related to the outcome (β = −.0004; *p* = .994). **Table 4** shows that the relationship between the covariates and CHLT-30 and REALM scores are similar in magnitude.

## Discussion

This CHLT-30 is useful as a tool for measuring cancer health literacy in both patients with cancer and people who have never had cancer. The psychometric properties of the CHLT-30 have already been documented in a cancer population (Dumenci et al., 2014; Fillon, 2015). The current study further expands the generalizability of the CHLT-30 beyond cancer populations. Results of this study strongly support the use of the CHLT-30 to measure cancer health literacy in people with no history of cancer. The fundamental psychometric properties of the CHLT-30 are remarkably similar in samples with and without cancer, lending strong support for the unidimensional measurement structure, invariant measurement properties between gender and race/ethnicity groups, high internal consistency, 2-week test-retest reliability, 6-month test-retest stability, and strong evidence for external validity.

Validation of the CHLT-30 to populations without cancer provides a research tool to understand the role of health literacy (as opposed to reading and writing literacy) in health behaviors ranging from decisions to engage in primary prevention to assisting cancer caregivers. The measurement of health literacy in behavioral intervention studies has become common. This instrument provides a needed tool and demonstrates the utility of disease-specific health literacy instruments. Understanding the role that cancer health literacy plays in health behaviors and health care decision-making will likely influence practices and recommendations about how we can influence screening behaviors and health outcomes and how to provide patients and their families with better support. This is especially important because 1.7 million people a year are expected to receive a cancer diagnosis, and most of them will have an actively involved family caregiver.

These results should pave the way to use the CHLT-30 in general populations for the purpose of identifying people at the lower end of the cancer health literacy continuum to devise intervention strategies to prevent negative health outcomes, particularly for those at high risk for developing cancer. For example, in a randomized clinical trial, Bell et al. (2016) reported that an educational intervention reduced unplanned health care use in people with low levels of health literacy. In another educational intervention study, Shah et al. (2015) showed improvements in diabetes control after a health literacy intervention. With the CHLT-30, people toward the lower end of cancer health literacy can be reliably identified and then targeted for intervention in future studies.

Results from external validation of the CHLT-30 showed that, after taking into account education, income, age, race/ethnicity, and gender, the CHLT-30 scores significantly predict the outcome (i.e., the self-confidence in making health decisions), whereas the REALM scores do not, and they showed that the correlation between the CHLT-30 and REALM is high (*r* = .734). Our results replicate the findings reported from the study using a cancer sample (Dumenci et al., 2014) and cast further doubts about the validity of REALM as a commonly used measure of health literacy (Dumenci et al., 2013).

Our study has some important limitations. Results from this study should not be generalized beyond non-Hispanic Whites and African Americans. Additionally, the CHLT-30 has not, to our knowledge, been used in educational interventions, but given the brief time required to complete and score the instrument (10 to 15 minutes), in our opinion the scale should be easy to use in intervention studies. When the administration time is prohibitive, the CHLT-6, a six-item test designed to identify people with limited cancer health literacy, should be considered as it takes only 3 to 4 minutes to administer and score. Further validation of the CHLT-30 is needed for racial/ethnic groups such as Hispanics and Asians. Also, our study showed that cancer health literacy can reliably be measured by the CHLT-30 in populations without cancer. This is a necessary condition to establish comparability of the CHLT-30 scores between cancer and groups without cancer. By establishing the measurement invariance (Meredith, 1993; Millsap, 2011), the cancer health literacy scores can be formally compared between the groups.

## Conclusion

The results presented in this study strongly support the validity and reliability of the CHLT-30 in populations without cancer. The CHLT-30 is easy to use in multiple settings. Clinicians and researchers interested in quantifying cancer health literacy in persons not diagnosed with cancer should be confident that the CHLT-30 measures cancer health literacy along a continuum and is particularly adept at measuring low cancer health literacy. The next steps would be to examine the CHLT-30 as a tool in a clinical setting.

## Figures and Tables

**Table 1:** Model Fit for the CHLT-30 by Gender, Race/Ethnicity, and Educational Attainment Groups

**Sample**	***N***	***X*^2^**	***df***	***p***	**RMSEA [90% CI]**	**CFI**	**TLI**
Female	315	446.74	405	.075	.018 [0, .028]	.982	.981
Male	197	457.47	405	.037	.026 [.007, .037]	.973	.971
Black	328	433.64	405	.157	.015 [0, .025]	.981	.979
White	180	437.05	405	.131	.021 [0, .034]	.972	.970
≤ High school education	205	425.44	405	.233	.016 [0, .030]	.963	.960
>High school education	307	424.83	405	.239	.013 [0, .024]	.984	.983
Combined	512	476.80	405	.008	.019 [.010, .025]	.985	.984

Note. CFI = comparative fit index; CHLT-30 = Cancer Health Literacy Test-30; CI = confidence interval; RMSEA = root mean square error of approximations; TLI = Tucker-Lewis index.

**Table 2:** The CHLT-30 Item Parameters

**Item**	**Item-Corrected**				
	**% Correct**	**Total *r^a^***	**Factor Loading**	**Difficulty**	**Discrimination**
1. High calorie	47	0.30	0.42	0.19	0.46
2. Next pill	87	0.45	0.72	−1.53	1.04
3. Chemotherapy	84	0.35	0.54	−1.85	0.64
4. Hemoglobin range	88	0.39	0.64	−1.82	0.83
5. Oral cancer	60	0.40	0.54	−0.48	0.64
6. Side effects	83	0.34	0.53	−1.82	0.62
7. Risk of complications	79	0.38	0.55	−1.47	0.66
8. Palliative care	62	0.41	0.55	−0.55	0.65
9. Biopsy	79	0.46	0.66	−1.19	0.89
10. Appointment location	88	0.37	0.61	−1.93	0.76
11. Body temperature	62	0.52	0.70	−0.42	0.98
12. Stage I cancer	76	0.45	0.64	−1.12	0.83
13. Direction	62	0.43	0.58	−0.51	0.71
14. Efficacy	76	0.33	0.47	−1.51	0.53
15. Tumor spread	61	0.36	0.48	−0.56	0.55
16. Generic drugs	70	0.39	0.54	−0.98	0.64
17. Survival rate	71	0.35	0.49	−1.12	0.56
18. Fasting	62	0.50	0.70	−0.45	0.97
19. Smoking risk	58	0.48	0.65	−0.32	0.85
20. Physical therapist	81	0.50	0.72	−1.24	1.04
21. Inoperable tumor	75	0.45	0.63	−1.05	0.82
22. High fiber food	86	0.31	0.49	−2.22	0.57
23. Metastasized	62	0.26	0.36	−0.86	0.38
24. Benign tumor	69	0.44	0.60	−0.81	0.74
25. Radiation treatment	87	0.43	0.70	−1.59	0.99
26. Complication rate	51	0.46	0.62	−0.05	0.79
27. Double dose	80	0.29	0.44	−1.96	0.49
28. Book chapter	77	0.44	0.66	−1.14	0.87
29. Dose time	52	0.37	0.50	−0.12	0.58
30. Map reading	84	0.42	0.65	−1.52	0.87

Note. CHLT-30 = Cancer Health Literacy Test-30.

a Corrected total correlation.

**Table 3:** Measurement Invariance Tests for the CHLT-30 Among Education, Race/Ethnicity, and Gender Groups

	**Invariance Test**			

**Groups**	**Fit Index**	**Configural**	**Scalar**	**Difference Test**

≤High school vs. >high school education	*X*^2^ (*df*)	850.11 (810)	898.05 (838)	47.09 (28)
	*P*	.159	.077	.013
	RMSEA	.014	.017	.001
	[90% CI]	[0, .023]	[0, .025]	–
	CFI	.978	.968	.010
	TLI	.977	.967	.010

White vs. Black	*X*^2^ (*df*)	869.58 (810)	886.63 (838)	25.11 (28)
	*P*	.072	.019	.622
	RMSEA	.017	.015	.001
	[90% CI]	[0, .026]	[0, .024]	–
	CFI	.975	.980	.005
	TLI	.973	.979	.005

Male vs. Female	*X*^2^ (*df*)	904.61 (810)	949.32 (838)	48.38 (28)
	*P*	.011	.004	.0002
	RMSEA	.021	.023	.002
	[90% CI]	[.011, .029]	[.014, .030]	–
	CFI	.978	.974	.004
	TLI	.976	.973	.003

Note: CHLT-30 = Cancer Health Literacy Test-30; CI = confidence interval; CFI = comparative fit index; RMSEA = root mean square error of approximations; TLI = Tucker-Lewis index.

**Table 4:** Correlations Between Health Literacy Scores and Covariates Estimated from Structural Equation Modeling

	**Health Literacy Scale**	
**Covariate**	**CHLT-30**	**REALM**
Education	.69	.61
Race/ethnicity (0 = White; 1 = Black)	−.53	−.40
Income	.40	.38
Gender (0 = male; 1 = female)	.10^*^	.05^*^
Age	−.07^*^	−.07^*^

Note. All correlation coefficients are significant (*p* < .01), unless marked with an asterisk. CHLT-30 = Cancer Health Literacy Test-30; REALM = Rapid Estimate of Adult Literacy in Medicine.
